# Statin use in patients with non‐HMGCR idiopathic inflammatory myopathies: A retrospective study

**DOI:** 10.1002/clc.23375

**Published:** 2020-05-20

**Authors:** Sangmee Sharon Bae, Buzand Oganesian, Ilana Golub, Christina Charles‐Schoeman

**Affiliations:** ^1^ Division of Rheumatology University of California Los Angeles Los Angeles California USA

**Keywords:** idiopathic inflammatory myopathy, muscle adverse events, retrospective study, statin use

## Abstract

**Background:**

Statins are the most widely used lipid lowering therapies which reduce cardiovascular risk, but are associated with muscular adverse events (AEs). Idiopathic inflammatory myopathies (IIM) are autoimmune diseases of the muscle with higher risk of cardiovascular disease. More data is needed regarding statin safety in patients with intrinsic muscle disease such as IIM.

**Hypothesis:**

Statins are tolerated in patients with IIM without leading to significant increase in muscular AEs.

**Methods:**

Statin use was retrospectively examined in a longitudinal IIM cohort. Safety analysis included assessment of muscular and nonmuscular AEs by chart review. IIM patients receiving a statin during the cohort follow‐up period were matched to IIM patients not receiving a statin for comparative analysis of longitudinal outcomes.

**Results:**

33/214 patients had a history of statin use. 63% started for primary prevention, while others were started for clinical ASCVD events, vascular surgery, IIM related heart failure, and cardiac transplantation. A high intensity statin was used in nine patients with non‐HMGCR myositis, and tolerated in 8/9 patients. Statin related muscular AE was noted in three patients. There were no cases of rhabdomyolysis, or statin related nonmuscular AEs in a median observation period of 5 years. In patients newly started on statins during cohort follow‐up (n = 7) there was no change in disease activity after statin initiation. Long term outcomes were not different between statin and nonstatin IIM control groups.

**Conclusion:**

Statins were well tolerated in patients with non‐HMGCR positive IIM. Given the accelerated atherosclerotic risk in IIM patients, further prospective studies of statin safety in IIM patients are warranted.

**Abbreviations**
AE: adverse events
DM: dermatomyositis
HMGCR: hydroxy‐3‐methylglutaryl‐coenzyme A reductase
IBM: inclusion body myositis
IIM: idiopathic inflammatory myopathies
IMNM: immune mediated necrotizing myopathy
MSA/MAA: myositis specific autoantibodies/myositis associated autoantibodies
PM: polymyositis

## 1 INTRODUCTION

Idiopathic inflammatory myopathies (IIM) are a group of autoinflammatory muscle diseases characterized by debilitating muscle weakness and increased morbidity and mortality. Patients with IIM have a significantly higher risk of cardiovascular (CV) disease and dyslipidemia compared to the general population.^1^, ^2^, ^3^ HMG CoA reductase inhibitors (statins) are the first line pharmacologic intervention for primary and secondary prevention of atherosclerotic cardiovascular disease (ASCVD).^4^, ^5^ Studies have consistently demonstrated the efficacy of statins on LDL cholesterol (LDL‐C) and reducing the risk of ischemic heart disease, stroke and CVD associated mortality.^6^, ^7^, ^8^ Data also suggests potential anti‐inflammatory benefits of statins in autoimmune diseases.^9^

Muscle adverse effects (AEs) including muscle pain, weakness and cramps are reported in 5% to 20% of patients on statins, and the majority of these resolve within weeks to months after drug cessation.^10^, ^11^ Rhabdomyolysis occurs with an incidence of approximately 0.4 per 10 000 patient years^12^ and has been reported mostly in patients with pre‐existing comorbid conditions or on multiple medications.^13^ Recently, a unique entity of statin induced immune mediated necrotizing myopathy (IMNM) has been described with autoantibodies targeting the HMG CoA reductase (HMGCR) protein.^14^, ^15^

Patients with underlying muscle disease including muscular dystrophies or metabolic myopathies are frequently hesitant to comply with statin therapy due to the widely publicized muscular AEs and small studies suggesting possible increased risk in these populations.^16^, ^17^ Such reports raise the concern of whether statins can be used safely for prevention of CV disease in these patients. To date, there is limited data on the safety and tolerability of statin use in IIM patients and further evaluation is warranted. Here we describe our experience of statin use in a longitudinal cohort of patients with IIM from a single tertiary academic center.

## 2 METHODS

### 2.1 Patients

A retrospective chart review was conducted of the UCLA IIM cohort, a longitudinal observational cohort including 214 adult patients with IIM. Patients fulfilled the EULAR/ACR classification criteria for IIM meeting the definition for at least “probable IIM.”^18^ All subjects gave written informed consent for the study under a protocol approved by the UCLA IRB (#10‐001833). All patients who reported ever taking a statin as part of their daily medications were identified.

Patients were also analyzed longitudinally if they were taking a daily statin at any point during their follow‐up period for >2 consecutive months and had available data regarding disease activity measures. Each patient receiving a statin during the cohort follow‐up period was matched to a control subject by (a) age ± 5 years, (b) gender, and (c) baseline physician global disease activity score by 100 mm visual analog scale (VAS) ±10 mm.^19^ Baseline visit was defined as the first visit on a statin for the statin group, and cohort enrollment visit for the control group.

All patients had baseline lipid profiles and repeat lipid profiles were routinely assessed during longitudinal follow up. The history of prior CV events was identified by questionnaires and chart review.

### 2.2 Atherosclerotic cardiovascular disease (ASCVD) risk assessment

We report 10 year ASCVD risk scores using the pooled cohort equations (PCE) risk calculator.^20^, ^21^ Outputs of the PCE risk calculator include the 10‐year and lifetime risk for developing a first CV event (nonfatal myocardial infarction, nonfatal stroke, fatal coronary heart disease, or fatal stroke). Patients with 10 year ASCVD risk of greater than 7.5% are recommended to initiate high to moderate intensity statin for primary prevention.

### 2.3 IIM disease assessments

Baseline disease characteristics were assessed including IIM type, myositis specific antibodies, and disease duration. Disease activity was assessed using physician global myositis disease activity by 100 mm VAS and 5 point Likert scales.^19^ Laboratory measures included creatine phosphokinase (CPK), aldolase, estimated sedimentation rate (ESR), and C‐reactive protein (CRP).

Disease activity was assessed at multiple time points. For patients that were already on a statin at time of cohort enrollment, disease activity was assessed at the baseline visit and the consecutive follow‐up visit. For patients that were started on a statin during the cohort follow‐up period, disease activity measures were collected at the visit before statin initiation and the first visit after statin initiation. Data from the most recent clinic visit was also reviewed to assess long term follow up. Clinically quiescent myositis was determined as no evidence of muscular or extra‐muscular myositis disease activity by subjective report, on physical exam and muscle enzymes.

### 2.4 Safety assessments

Patients in the cohort were followed every 2 to 3 months in clinic. Data from all visits were reviewed for the following prespecified muscular and nonmuscular AEs: (a) myalgias, (b) CPK elevations (>25% increase compared to prior visit, for two consecutive visits), (c) rhabdomyolysis (new CPK elevation of >10X ULN), (d) elevated liver enzymes (elevation in gamma‐glutamyl transferase and transaminases above ULN in the setting of normal CPK, for two consecutive visits), (e) GI intolerance (eg, nausea, vomiting, abdominal cramps), (f) worsening renal function (increased creatinine >50% from baseline), and (g) discontinuation of statin. AEs were determined as statin‐related if (a) there was a temporal relation with statin initiation, dose escalation, or change in statin agent in the absence of other clinical causes or (b) the event lead to statin discontinuation with subsequent improvement in signs/symptoms.

### 2.5 Statistical analysis

Baseline demographics and disease activity measures between groups were compared using chi‐square test for categorical variables, and student's *t*‐test or Wilcoxon Rank sum test for continuous variables. In comparing changes in disease activity measures, paired student's *t*‐test and paired Wilcoxon signed rank test were used. Statistical significance was defined as a two‐sided *P* value of <.05. Statistical analysis was performed on JMP Pro version 13.0.0 (SAS Institute Inc., Cary, North Carolina).

## 3 RESULTS

### 3.1 Statin use in the IIM cohort

**FIGURE 1:**
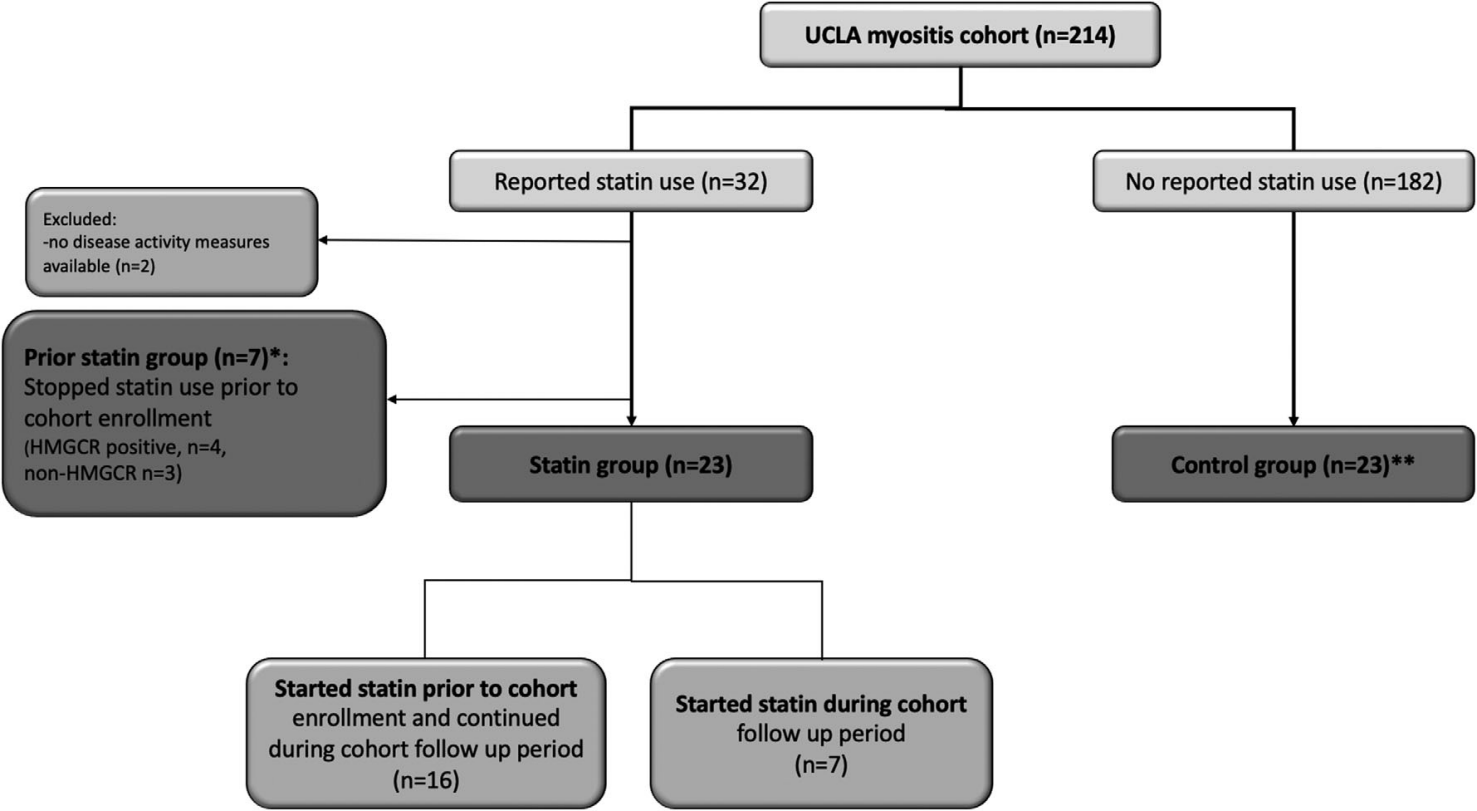
Flowchart of patient groups. *Patients that discontinued statin prior to cohort enrolment. **Control group: matched to each patient in statin group by (a) age ± 5 years, (b) gender, and (c) baseline physician global disease activity score by 100 mm visual analog scale (VAS) ±10 mm

Past or present statin use was identified in 33 patients in the IIM cohort (Figure 1). Seven patients reported statin use in the past but had discontinued the statin prior to cohort enrollment. Twenty‐three patients were actively receiving a statin during the cohort follow‐up period with disease activity measures available for review (statin group, Table 1). These patients were matched to IIM controls by age, gender and myositis disease activity (control group, see Section 2 for details).

**TABLE 1:** Baseline demographics and ASCVD risk for statin group (n = 23)

ID	Age	Sex	Race	Ethnicity (Hispanic)	IIM type	MSA/MAA	Disease duration (months)	CPK (U/L)^a^	Disease activity VAS (0‐100 mm)	Disease activity Likert (0‐4)	History of ASCVD	10 y ASCVD risk (%)	Total cholesterol	LDL	HDL	Smoking
1	75	F	White		PM	NT	379	29	17	1		26.4	277	182	73	None
2	59	F	White		DM	negative	522	108	25	1		6.7	189	104	54	None
3	78	M	Asian		IBM	NT	85	475	40	2	AAA s/p repair	70.1	135	48	41	None
4	62	M	White	Hispanic	DM	NT	96	113	80	3	CAD	20.2	181	101	52	None
5	51	M	White		DM	negative	165	137	50	2		4.7	237	111	57	None
6	64	F	White		DM	NT	241	28	80	3		10.7	152	58	30	None
7	71	M	White		IBM	NT	75	607	50	2		21.1	169	76	54	None
8	75	F	White		DM	NT	427	566	50	2	CAD	24.5	184	110	60	Former
9	62	F	White		DM	NT	84	112	60	2		4.0	173	91	61	None
10	47	M	White		DM	negative	65	109	80	3		4.2	181	111	38	Former
11	43	F	Asian		DM	MDA5	1	33	90	4		1.5	202	93	43	None
12	54	F	White		DM	jo1	44	41	75	3		1.5	195	95	34	None
13	28	F	White		DM	p155/140	2	64	70	3	TIA/lacunar infarct	0.3	133	72	60	None
14	73	F	White		DM	indRo	255	58	12	1	CVA	18.2	184	101	65	None
15	62	M	White		DM	indRo	67	75	59	2	CAD	11.7	158	89	45	Former
16	67	F	Black		DM	MJ, U1RNP	416	30	50	2	CVA,	32.5	150	63	74	None
17	34	M	Asian		DM	unidentified ab	98	214	8	1		2.6	274	175	55	None
18	58	F	White		DM	MJ	75	53	9	1	CAD, carotid atherosclerosis	4.1	305	207	72	None
19	61	M	Black		PM	negative	170	267	5	1		16.1	248	196	52	None
20	69	F	White		DM	p155/140	8	100	45	2		6.8	216	89	107	None
21	55	F	White		DM	indRo	128	45	30	1	CHF	3.0	259	127	69	Former
22	38	M	Black	Hispanic	DM	Ku, indRo	74	1369	50	2	CHF, cardiac transplant	6.0	174	53	42	None
23	51	F	White		DM	NT	178	57	30	1	carotid atherosclerosis	3.7	247	188	44	None

Abbreviations: AAA, abdominal aortic aneurysm; CAD, coronary artery disease; CVA, cerebral vascular accident; DM, dermatomyositis; HTN, hypertension; indRo, indeterminate Ro; IBM, inclusion body myositis; MSA/MAA, myositis specific antibodies/myositis associated antibodies; NT, not tested; PM, polymyositis.

a Normal range: female 26 to 192, male 39 to 308.

### 3.2 Indications for statin therapy

Among the 33 patients, 9 patients (27%) were on a statin for a history of clinical ASCVD; coronary artery disease with revascularization (n = 6), stroke (n = 2) and transient ischemic attack (n = 1) (Table 2). Two DM patients had NYHA class III/IV heart failure related to their DM, one of which was started on a statin after cardiac transplantation. One patient was on a statin for abdominal aortic aneurysm. Most patients (21/33) were started on a statin for primary prevention given their increased risk of CVD with the presence of hypertension, diabetes, and/or dyslipidemia.

**TABLE 2:** Statin treatment: agents, dose, indication for use and reported AEs during statin use

ID^a^	Agent	Dose (mg/d)	Indication	Duration on statin after IIM diagnosis (months)	AEs (presumed cause^b^)
1	Atorvastatin/Pravastatin/Rosuvastatin	20/80/5	HLD	61	Elevated liver enzymes (PBC flare), nausea (unknown), diarrhea (unknown)
2	**Rosuvastatin** ^c^	**20**	HTN, diabetes	70	Nausea(MMF)
3	Simvastatin	40	Diabetes, HTN, AAA s/p repair	90	Elevated liver enzymes (antibiotics), elevated Cr (hypovolemia)
4	Lovastatin/**Atorvastatin** ^c^	40/**40**	CAD s/p PCI	99	
5	**Rosuvastatin** ^c^	**20**	HLD	108	
6	Pravastatin	20	HLD	90	Elevated liver enzymes (MMF)
7	Rosuvastatin	10	HLD	27	
8	Atorvastatin	10	CAD, HLD, HTN	21	
9	Atorvastatin	10	HLD	84	
10	Rosuvastatin	10	HTN	71	Tendonitis (unknown), abdominal pain (interstitial cystitis)
11	Atorvastatin	10	diabetes, HTN	2	Elevated liver enzymes/renal failure (hemorrhagic shock)
12	Atorvastatin	20	HLD	47	Abdominal cramps (cyclophosphamide)
13	Atorvastatin	10	TIA/lacunar infarct	7	Diarrhea (unknown), dizziness (MMF), **Myalgia (statin)**
14	**Atorvastatin** ^c^	**40**	CVA with carotid artery occlusion	77	Diarrhea(MMF)
15	**Atorvastatin** ^c^	**40**	CAD s/p PCI	30	
16	**Atorvastatin** ^c^	**40**	CVA, DM, HTN	42	
17	Rosuvastatin	5	HLD	27	
18	**Rosuvastatin** ^c^	**40**	HLD, CAD, carotid atherosclerosis	43	
19	Rosuvastatin	5	HTN, HLD	3	Tendonitis
20	Atorvastatin	10	HLD	9	
21	Atorvastatin	10	cardiomyopathy, CHF	29	Elevated liver enzymes/nausea (cyclophosphamide)
22	Pravastatin	20	cardiomyopathy s/p transplant	32	Muscle spasms of neck/abdomen (post heart transplant, surgical site complication)
23	Atorvastatin	20	HLD	48	Abdominal pain(biliary colic)
24	Atorvastatin	5/10	N/A	14	
25	Atorvastatin	N/A	N/A	0	Myalgia **(statin)**
26	Simvastatin/**Pravastatin**	40/**80**	HLD	0	Myalgia, weakness **(simvastatin)**
27	Atorvastatin	10	CAD, diabetes, HTN	0	Onset of necrotizing myopathy
28	Atorvastatin	N/A	N/A	0	Onset of necrotizing myopathy
29	**Atorvastatin** ^c^	**40**	Diabetes, HTN, HLD	0	Onset of necrotizing myopathy
30	Atorvastatin	20	Diabetes, HTN, HLD	0	Onset of necrotizing myopathy
31	Atorvastatin	20	CVD, Diabetes	N/A	N/A
32	Atorvastatin	N/A	HLD	N/A	N/A
33	**Atorvastatin** ^c^	**40**	HLD	N/A	N/A

Abbreviations: AEs, adverse events; MMF, mycophenolate mofetil; N/A, data not available; PBC, primary biliary cirrhosis.

a Patients 24 to 30 are in prior statin group, 31 to 33 are patients without follow‐up data (excluded from statin group for lack of disease activity assessment).

b Presumed cause: based on temporal correlation of adverse event with onset or dose/change of medication or clinical event.

c High intensity statin include Atorvastatin 40 to 80 mg, rosuvastatin 20 to 40 mg.

Bold values are high intensity statin.

The mean 10‐year ASCVD risk score calculated at baseline visit for the statin group was 13.1 (0.3‐70.1) mean (range) (Table 1), which was numerically higher compared to the matched nonstatin control group (11.8 [0.2‐45.9], mean [range], *P* = .77). 10/23 patients in the statin group and 8/23 patients in the control group had high ASCVD risk (10 year risk >7.5%).

### 3.3 Type of statin therapy

The most common type of statin used was atorvastatin 5 to 40 mg (n = 22) followed by rosuvastatin 5 to 20 mg (n = 8) (Table 2). Simvastatin was used in two patients, and one reported related myalgias. Simvastatin has been associated with a higher risk of muscular AEs compared to other statins.^11^ A high intensity statin was used in nine patients with non‐HMGCR myositis, and tolerated in 8/9 patients. The majority of these patients were started after a clinical ASCVD event.

### 3.4 Statin safety

AEs during statin therapy are outlined in Table 2. Seven patients were previously on statins but discontinued prior to myositis diagnosis (*prior statin group* in Figure 1). Four (57%) patients discontinued statins due to a new diagnosis of HMGCR antibody positive necrotizing myositis. At the time of disease onset, all four patients had been on statins at a stable dose for at least 1 year (median (range) of 4 (1‐10) years). The remaining three patients were later diagnosed with DM. Two patients had discontinued statins due to muscle AEs that resolved within 3 to 6 months after discontinuation of statins. Both patients were diagnosed with IIM >3 years after their last episode of statin related muscle AE. The third patient tolerated statin but discontinued when she began chemotherapy for lung cancer.

Among the 23 patients in the statin group, one patient (pt 13) developed statin related myalgia which lead to discontinuation of statin (Table 2). No other statin‐related muscular AEs occurred in the remaining 22 patients. Four other patients either switched or discontinued statin therapy, none of which were due to statin related AEs. There was one patient (pt 4) who switched lovastatin to high intensity atorvastatin after a myocardial infarction. The remaining patients (18/23) had no change in dose or type of statin therapy during the total observation period of 65 (4‐106) months, median (range).

The most common laboratory abnormality was elevation in liver enzymes (n = 5), followed by increased creatinine (n = 2), none of which were statin‐related (Table 3). Other AEs included nausea (n = 3), diarrhea (n = 3), abdominal pain/cramps (n = 4), and tendonitis (n = 2), all of which resolved without change in statin therapy.

**TABLE 3:** Statin group vs matched control group

	Statin group (N = 23)	Control group (N = 23)	*P* value
*Age (yrs)*, mean (SD)	58.19 (12.75)	58.69 (14.02)	.89
*Gender (female)*, N (%)	14 (60.87)	14 (60.87)	1.00
*Race (White)*, N (%)	17 (73.91)	18 (78.26)	.89
*Ethnicity (Hispanic)*, N (%)	2 (8.70)	4 (17.39)	.37
*IIM type*, N (%)			.59
Dermatomyositis	19 (82.61)	18 (78.26)	
Polymyositis	2 (8.70)	4 (17.39)	
Inclusion body myositis	2 (8.70)	1 (4.35)	
*MSA/MAA*, N (%)			.43
Antisynthetase ab	1 (4.35)	4 (17.39)	
Other MSA/MAA	9 (39.13)	7 (30.43)	
None	5 (21.74)	3 (13.04)	
Not tested	8 (34.78)	9 (39.13)	
*Disease duration (months)*	105.39 (142.80)	63.65(106.43)	.37
*Medications*, n (%)			
Prednisone	14 (61)	13 (57)	.48
Daily prednisone dose	12 (15)	21(29)	.20
Number of immunomodulatory drugs other than steroids, median (range)	1 (0‐3)	1 (0‐3)	.60
*Lipid profile*			
Total cholesterol (mg/dL)	198.22 (56.66)	210.52 (36.90)	.39
LDL‐C (mg/dL)	112.87 (49.12)	124.95 (35.82)	.35
HDL‐C (mg/dL)	54.82 (18.03)	60.35 (27.66)	.43
Triglycerides (mg/dL)	178.09 (105.61)	169.91 (127.71)	.79
*Baseline disease activity*			
Physician global VAS (mm)	46.30 (25.89)	38.04 (20.07)	.23
Physician global Likert, median (IQR)	2 (1‐3)	2 (1‐3)	.40
CPK (U/L)	203.91 (305.51)	204.41 (309.29)	.67
Aldolase (U/L)	6.83 (3.30)	6.07 (1.11)	.46
ESR (mm/h)	26.25 (15.81)	26.8 (21.52)	.92
CRP (mg/dL)	0.57 (0.35)	0.66 (1.07)	.70
*Change (∆) in disease activity* ^a^			
∆ Physician global activity VAS (0‐100 mm)	6.74 (15.38)	1.60 (13.1)	.50
∆ Physician global activity Likert	−0.26 (0.45)	−0.13 (0.46)	.33
∆ CPK (U/L)	−7.95 (86.09)	60.05 (308.23)	.55
∆ Aldolase (U/L)	0.09 (3.00)	−0.98 (4.90)	.55
∆ ESR (mm/h)	5.06 (14.07)	0.69 (26.00)	.54
∆ CRP (mg/dL)	−1.19 (2.54)	−0.19 (1.41)	.20
Follow‐up interval, median (range)	4 (1‐60) months	3 (1‐12) months	.16

*Note:* Values are mean (SD) unless specified otherwise.

Abbreviations: CPK, creatine phosphokinase; CRP, C‐reactive protein; ESR, estimated sedimentation rate; VAS, visual analog scale.

a Change in disease activity measures between two consecutive visits.

### 3.5 Statin efficacy

In patients newly started on a statin therapy during the cohort follow‐up (n = 7), statins effectively lowered LDL by 44.3 (54.5) mg/dL (*P* = .04) and also increased HDL by 12.3 (11.3) mg/dL (*P* = .06) over 20.0 (16.9) months on statin therapy, mean (SD) for all. In other patients maintained on statin therapy during the follow‐up period (n = 16), LDL levels remained stable (94 (31) mg/dL;[baseline visit], 96 (49) mg/dL;[most recent follow‐up visit], mean (SD), p = NS) suggesting compliance with statin use. Duration of reported statin use after IIM diagnosis was 61 (2‐108) months, median (range).

### 3.6 Longitudinal analysis: baseline characteristics of statin and comparator groups

Comparison of statin (n = 23) and control groups (n = 23) is outlined in Table 3. There were no differences between the groups in IIM type, autoantibody subgroups, disease duration, medications, IIM disease activity at the baseline visit. Although disease duration varied from 1 month to over 40 years, most patients (19/23) had chronic myositis of >3 years. 17/23 patients had low to moderate physician global disease activity scores with mean CPK levels in the normal range. Baseline lipid profiles were similar between the two groups.

### 3.7 longitudinal analysis: disease activity assessments

To assess whether statin use was associated with worsened myositis activity, changes in disease activity between baseline and consecutive follow‐up visits were compared between statin and control groups. Consecutive follow‐up visit was chosen for repeat disease activity assessment to minimize potential confounding by changes in immunomodulatory medications. Changes in disease activity measures over time were not significantly different between patients on statins and IIM controls (p = NS for all, Table 3).

Subgroup analysis of patients who were newly started on a statin during cohort follow‐up (n = 7) and matched controls showed no differences in disease activity measures after statin initiation. There were no significant changes in IIM disease activity measures or assessments of inflammation (p = NS for physician global VAS, CPK, aldolase, ESR, CRP; Table S1). The interval between the two visits was short at 3 (1‐4) months, median (range).

For long term follow‐up, we reviewed data from the most recent clinic visits. The median (range) follow‐up time was 65 (4‐106) months in the statin group and 75 (8‐130) months in the control group, (*P* = .07). Patients in both groups were on similar number of immunomodulatory medications in order to control the disease (2[0‐3] in statin group vs 1[0‐3] in control group, mean[range], *P* = .7), and similar doses of daily prednisone (7[10]mg/day in statin group vs 3 [5] mg/day in control group, mean [SD], *P* = .11). Both groups had nine patients with clinically quiescent myositis.

## 4 DISCUSSION

The increased risk of accelerated atherosclerosis and CVD in IIM patients is well recognized as it is in other chronic rheumatic diseases such as RA and systemic lupus erythematosus.^22^, ^23^ Studies show an increased risk of myocardial infarction and stroke in IIM patients compared to the general population,^1^, ^2^ as well as a higher proportion of traditional CV risk factors such as hypertension, diabetes, obesity and dyslipidemia.^24^ Histopathologic studies also implicate direct involvement of the microvasculature in the pathophysiology of DM.^25^, ^26^ Taken together, this data suggests that the study of lipids and lipid lowering agents is particularly relevant to patients with inflammatory myopathies.

Statins are the first line lipid lowering agent and make up over 80% of all lipid lowering medications used in clinical practice.^27^ Muscular AEs are reported in 5% to 20% of the general population using statins^28^ and in the current study occurred in 10% (3/29) of IIM patients without HMGCR antibody‐associated disease, which is within the range of AEs in the general population. This data is also consistent with the survey study in which IIM specialists reported worsening muscle symptoms in ~10% of their IIM patients using statins, with the majority improving after discontinuation of statin therapy.^29^

In contrast to the widely publicized concerns of statin related muscular AEs, a systematic review of clinical trials reported that muscular AEs were minimally higher in statin patients when compared to placebo controls,^30^ suggesting that statin related muscular complaints may be overemphasized in clinical practice. The purpose of this study was to further extend the understanding of statin related muscular AEs, by examining outcomes in a group of patients with intrinsic auto inflammatory muscle diseases.

The current work reported statin use in IIM patients from a longitudinal cohort at a tertiary academic center, which included high risk, complex patients with a history of clinical ASCVD events, vascular surgery, IIM related heart failure and cardiac transplantation. To assess the impact of statins on myositis disease activity, we compared IIM patients on statins to a matched nonstatin exposed IIM group with median follow‐up of over 5 years. Recent work by Borges and colleagues also reported a retrospective analysis of statin use in 24 patients with IIM on either atorvastatin or simvastatin with a slightly shorter median follow‐up of 22.5 months.^31^ While this previous study did not include an IIM comparator group, or high risk CVD patients (such as patients with prior ASCVD event), a similarly good tolerability and safety of statins was observed in IIM patients with stable disease.

The ASCVD risk assessment tool was developed as a strategy to personalize the estimation of ASCVD risk in order to help target CV preventative strategies, including statin use.^32^ The ACC/AHA PCE risk calculator is widely used to personalize the estimation of benefits from risk reducing therapies. However, there are caveats to the application of the risk calculator that are noteworthy when considering statin therapy in patients with IIM as presented in our study.

First, the risk calculator is *not* recommended to be used in patients with known prior ASCVD events, as statin use should be considered for these patients regardless of age, gender or other risk factors.^4^, ^33^ In our current study, there were 10 IIM patients in the statin group that had prior clinical ASCVD events or other high risk features including heart failure, postcardiac transplant, and post‐vascular surgery. These patients were appropriately started on a statin regardless of their ASCVD scores. Statin therapy was well tolerated in 9/10 patients (all except 1 patient with statin related myalgias).

Second, the ACC/AHA guideline highlights that clinical judgment and consideration of each individual's conditions remains important when deciding on a management plan. In the current analysis, mean ASCVD risk scores were similar between the statin and nonstatin IIM patients, with the latter group including eight patients who had an increased 10 years ASCVD risk of over 7.5% but no statin use. In patients with IIM, weighing the risks of statin related muscular AEs against the CV benefits has previously been difficult due to no studies of statin tolerability in IIM patients prior to the study by Bourges and the current work.

The use of lipid lowering therapies including statins in patients with underlying muscle disease has remained an area of debate. Small case studies have reported severe muscular complications with lipid lowering therapies in patients with metabolic myopathies,^16^, ^34^ and a cross‐sectional study of patients with lipid‐lowering drug‐induced myopathies, reported a higher prevalence of underlying metabolic muscle diseases than expected in the general population.^17^ Conversely, recent studies have demonstrated improved muscular function in muscular dystrophy models with simvastatin, suggesting positive effects of statin therapy.^35^

In our experience, lipid‐lowering therapy is often held in patients presenting with a new diagnosis of myositis due to concerns that the therapy may have a negative impact on the muscle disease. Case reports have also described potential associations between statin use and the onset of DM or PM.^36^, ^37^, ^38^, ^39^, ^40^ We did not find this association in the current work. Two patients who had used statins prior to cohort enrollment reported statin intolerance and later developed DM. However, the statin‐related muscle symptoms had resolved at least 3 years prior to DM disease onset. Work by Mamyrova and colleagues has described associations between environmental exposures including sun exposure, infections and certain medications (anti‐hypertensives, anti‐depressants, NSAIDs) with flares of DM,^41^ but no associations with lipid‐lowering therapy were noted in that study.

Four patients in our study had HMGCR positive necrotizing myopathies with a history of statin use prior to onset of the muscle symptoms. IMNM is a recently defined autoimmune myopathy associated with autoantibodies targeting the HMGCR protein. The majority of these cases have been associated with prior statin use, although 37% of patients in the initial cohort did not have a history of statin exposure.^14^ It should be noted that this condition is extremely rare with an estimated incidence of two cases per million people per year.^42^ No routine screening for HMGCR antibodies prior to statin use is currently recommended in the general population or for patients with other known types of IIM.

The type and intensity of statin therapy has been shown to affect tolerability. Studies in the general population have reported approximately 1.5 to 2 times the rate of treatment related AEs leading to drug discontinuation in patients on high‐intensity statins compared to patients on low to moderate‐intensity statins.^43^, ^44^ In the current study 31% (9/29) of non HMGCR IIM patients received high intensity statin therapy which was tolerated in 8/9 patients (all except 1 patient with statin related myalgias). This suggests that high intensity statins can be considered in non‐HMGCR IIM patients when clinically indicated. Among different statin types, atorvastatin and pravastatin have been shown to have lower statin associated muscular AEs and be better tolerated compared to simvastatin.^4^, ^11^ Most IIM patients in the current study were placed on atorvastatin or rosuvastatin for moderate to high intensity therapy, and pravastatin for low to moderate intensity therapy. However, in the study by Borges and colleagues, which also reported statin tolerability in IIM patients, 50% of patients received simvastatin without evidence of muscular AEs.

The timing of statin initiation may be of clinical importance. The majority of IIM patients starting statin therapy or continuing statin therapy during the follow‐up period of the current study had chronic myositis of several years' duration and low disease activity. For patients with higher disease activity, lower intensity statins such as pravastatin or lower doses of atorvastatin and rosuvastatin were used, unless patients had an ASCVD event. One patient, in particular, had severe cardiac involvement and DM‐associated inflammation documented on pathology of the explant heart at the time of statin initiation.^45^ This patient did well clinically following initiation of statin therapy despite active muscle disease at initiation.

Finally, in addition to CV benefits, statins have been shown to have beneficial effects on multiple inflammatory pathways.^46^ The trial of atorvastatin in RA demonstrated significant improvement in disease activity scores when atorvastatin 40 mg was added to existing disease modifying rheumatic agents in active RA patients.^47^ Similar anti‐inflammatory effects of statins have been demonstrated in inflammatory vasculitides.^48^ Interestingly, the current work demonstrated a modest trend for greater decreases in CRP levels in the statin group compared to the control group. This has previously been reported with statin therapy in the general population.^49^ Additional work to examine the molecular effects of statins on disease pathogenesis in IIM may be warranted.

Our study has several limitations. IIM are rare diseases with a prevalence of 2 to 58 per 100 000.^50^ Therefore, the total number of patients reported in the current work is small. However, all patients were part of the same single center cohort of over 200 IIM patients and statin patients were compared to matched, nonstatin exposed IIM controls from the same cohort. The majority of patients presented in this work had DM, which is consistent with the predominance of DM in our center's longitudinal cohort. Additional study of statin tolerance in larger numbers of patients with other IIM is warranted. In addition, limitations to a retrospective review include other selection bias of the patients included in the initial cohort and the possibility of data gaps including lack of proper adverse event (AE) recording and medication compliance. Many patients initiated statin use under the care of an outside physician prior to referral to our center, and thus missing data was inevitable. Also, with the concern of muscular AEs, many patients with high disease activity may not have been treated with a statin which may have introduced a potential bias to the statin cohort reported. However, all patients were followed routinely in our clinic, and chart data was carefully reviewed in order to limit these known caveats. Lastly, a median follow‐up period of 5 years may be inadequate to determine long term safety. Longer term follow‐up of this cohort is ongoing.

In conclusion, statins were well tolerated in a single center retrospective study of IIM patients. Use of statins may be considered in IIM patients without HMGCR antibody‐associated IMNM when clinically indicated for CV risk reduction. Further prospective studies with larger patient groups are warranted to assess the safety of statins in IIM patients.

## CONFLICT OF INTEREST

All other authors have neither conflicts of interest nor conflicts relating to financial support or other benefits from commercial sources for the work supported in this manuscript.

## AUTHOR CONTRIBUTIONS

All authors take responsibility for all aspects of the reliability and freedom from bias of the data presented and their discussed interpretation

## Supporting information


**Table S1** Change in disease activity measures in patients newly started on statin. Mean (SD) unless specified otherwise. Abbreviations: CPK, creatine phosphokinase; CRP, C‐Reactive protein; ESR, estimated sedimentation rate; VAS, visual analogue scale
