# Supplementary material for: Statin use in patients with non‐HMGCR idiopathic inflammatory myopathies: A retrospective study
Source: Clin Cardiol. 2020 May 20;43(7):732–42. doi: 10.1002/clc.23375 (PMC7368310; doi:10.1002/clc.23375)
Supplement: Supplementary file 1 — Table S1 Change in disease activity measures in patients newly started on statin. Mean (SD) unless specified otherwise. Abbreviations: CPK, creatine phosphokinase; CRP, C‐Reactive protein; ESR, estimated sedimentation rate; VAS, visual analogue scale [file CLC-43-732-s001.docx]

**Supplementary table**

| **Disease activity measures** | **Before statin**  (N=7) | **After statin**  (N=7) | **P value** |
| --- | --- | --- | --- |
| Physician global VAS (0-100 mm) | 30.71 (23.44) | 31.71 (17.93) | 0.89 |
| Physician global Likert, Median (Range) | 1 (1-2) | 1 (1-2) | 0.36 |
| CPK (U/L) | 392.57(654.41) | 300.71 (478.94) | 0.23 |
| Aldolase (U/L) | 8.67 (7.24) | 7.16 (4.65) | 0.34 |
| ESR (mm/hr) | 23.29 (14.06) | 23.00 (15.56) | 0.97 |
| CRP (mg/dL) | 1.27 (1.14) | 0.53 (0.53) | 0.12 |
| Follow up interval, Median (range) | 3 (1-4) months | | |

**Change in disease activity measures in patients newly started on statin**

Mean (SD) unless specified otherwise.

Abbreviations: VAS: Visual Analogue Scale, CPK: creatine phosphokinase, ESR: estimated sedimentation rate, CRP: C-Reactive protein
